# Plasmid DNA gene therapy of the Niemann-Pick C1 mouse with transferrin receptor-targeted Trojan horse liposomes

**DOI:** 10.1038/s41598-020-70290-w

**Published:** 2020-08-07

**Authors:** Dahai Jiang, Hungyen Lee, William M. Pardridge

**Affiliations:** The Lipogene Company, 2649 Townsgate Road, Thousand Oaks, CA 91361 USA

**Keywords:** Biotechnology, Neuroscience

## Abstract

Niemann-Pick C1 (NPC1) is a lysosomal cholesterol storage disorder, that severely affects the brain, and is caused by mutations in the NPC1 gene, which encodes an intracellular membrane transporter of non-esterified cholesterol. Therapeutic options for NPC1 are few, and classical enzyme replacement therapy with the recombinant protein is not possible as the NPC1 gene product is an insoluble membrane protein, which increases the need for development of gene therapy for NPC1. While viral based gene therapy is under development, it is important to investigate alternative approaches to brain gene therapy without viral vectors. The present work develops a plasmid DNA approach to gene therapy of NPC1 using Trojan horse liposomes (THLs), wherein the plasmid DNA is encapsulated in 100 nm pegylated liposomes, which are targeted to organs with a monoclonal antibody against the mouse transferrin receptor. THLs were encapsulated with a 8.0 kb plasmid DNA encoding the 3.9 kb human NPC1 open reading frame, under the influence of a 1.5 kb platelet derived growth factor B (PDGFB) promoter. THLs were administered weekly beginning at 6–7 weeks in the NPC1^−/−^ null mouse, and delivery of the plasmid DNA, and NPC1 mRNA expression in brain, spleen, and liver were confirmed by quantitative PCR. THL treatment reduced tissue inclusion bodies in brain, and peripheral organs, but did not prolong lifespan in these mice. The work suggests that early treatment after birth may be required to reverse this disease model with NPC1 gene replacement therapy.

## Introduction

Niemann-Pick C1 (NPC1) disease is an inherited lysosomal storage disorder caused by mutations in the gene encoding the NPC1 protein, which is a 200 kDa intracellular membrane transporter of non-esterified cholesterol. Cell cholesterol accumulation leads to intracellular inclusion bodies, which cause neurodegeneration, and hepatosplenomegaly^1^. The diseases causes early mortality in most cases, and is associated with dementia, seizures, ataxia, and decreased audition^1^. There is no approved therapy for NPC1. New treatments may be tested in the NPC1 null mouse (NPC1^−/−^) such as the NPC1^*m1N*^ mouse^2,3^, and the systemic administration of large doses of hydroxypropyl beta cyclodextrin (HPβCD) to NPC1^−/−^ mice prolongs lifespan^4^. HPβCD does not cross the blood–brain barrier (BBB)^5^, and HPβCD administration to NPC1 patients uses intrathecal administration via injections into the lumbar cerebrospinal fluid (CSF)^6^. However, intrathecal drug delivery to brain only allows for drug exposure at the CSF surface of the brain^7^, and intrathecal HPβCD has not been approved. NPC1 gene therapy with adeno-associated virus (AAV) serotypes, e.g. AAV9, is possible, particularly with self-complementary AAV (scAAV), as these serotypes cross the BBB^8^. However, the maximal size of the expression cassette that can be inserted in the scAAV is < 2.3 kb^9^, and the size of the NPC1 open reading frame alone is 3.9 kb. Single stranded AAV (ssAAV) traverses the BBB less efficiently^9,10^, but this viral genome will accept expression cassettes as large as 4.7 kb^9^.

An alternative approach to NPC1 gene therapy is the use of Trojan horse liposomes (THLs). THLs are formed by encapsulation of non-viral plasmid DNA in the interior of 100–150 nm pegylated liposomes, which are targeted with a receptor-specific monoclonal antibody (MAb)^11^. The MAb targets a receptor expressed on the BBB, such as the transferrin receptor (TfR). The TfRMAb is conjugated on the surface of the THL and acts as a molecular Trojan horse to ferry the liposome-encapsulated plasmid DNA across both the BBB and the brain cell plasma membrane, followed by delivery of the plasmid DNA to the nuclear compartment^12,13^. Plasmid DNAs as large as 22 kb can be encapsulated in THLs, and genes encoded in such large plasmid DNAs are expressed in vivo in the brain following IV administration of THLs^14^. Therefore, a large therapeutic gene such as NPC1 can be placed under the influence of a large promoter that is specific for neurons. One such neuron-selective promoter is 1.5 kb of the 5′-flanking sequence of the human platelet derived growth factor-B (PDGFB) gene^15^. The PDGFB promoter enables high transgene expression in the brain in vivo^16,17^, and produces a higher degree of transgene expression in neurons as compared to the cytomegalovirus (CMV) promoter^18^.

In the present investigation, 6 week old NPC1^*m1N*^ mice were treated with weekly IV administration of either vehicle or TfRMAb targeted THLs encapsulating a 8 kb expression plasmid DNA encoding the 1.5 kb PDGFB promoter and the 3.9 kb human NPC1 open reading frame. The IV injection dose was 6 μg plasmid DNA per mouse, which was shown by quantitative PCR to deliver multiple copies of the plasmid DNA per brain cell.

## Results

### Bioactivity of pPDGFB-NPC1 plasmid DNA, recombinant TfRMAb, and THL stability at 4 °C

**Figure 1:**
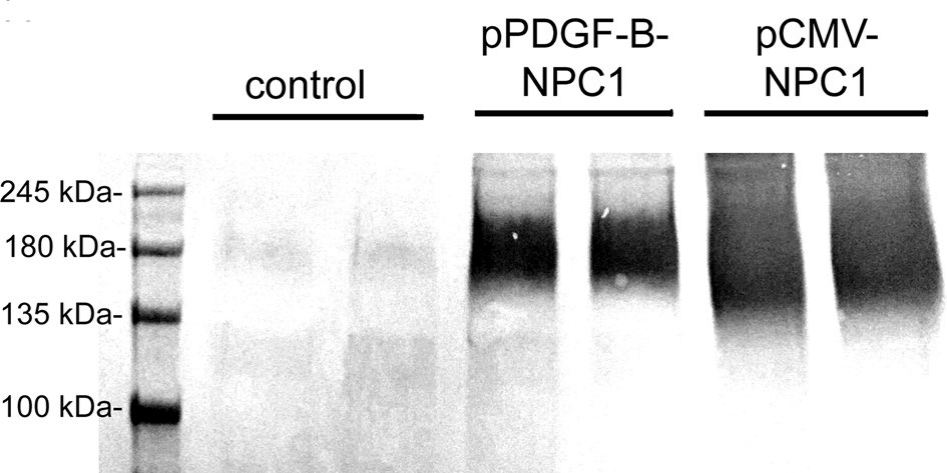
Human NPC1 Western blot of COS cell lysates removed 2 days following cell lipofection with Lipofectamine 2000 and 2 μg DNA per dish of either no plasmid (control), pPDGF-B-NPC1 plasmid DNA, or pCMV-NPC1 plasmid DNA. Duplicate lanes of each are shown. Lipofection with either NPC1 expression plasmid produces a 180–200 kDa immunoreactive protein, which corresponds with the size of the human NPC1 protein.

**Figure 2:**
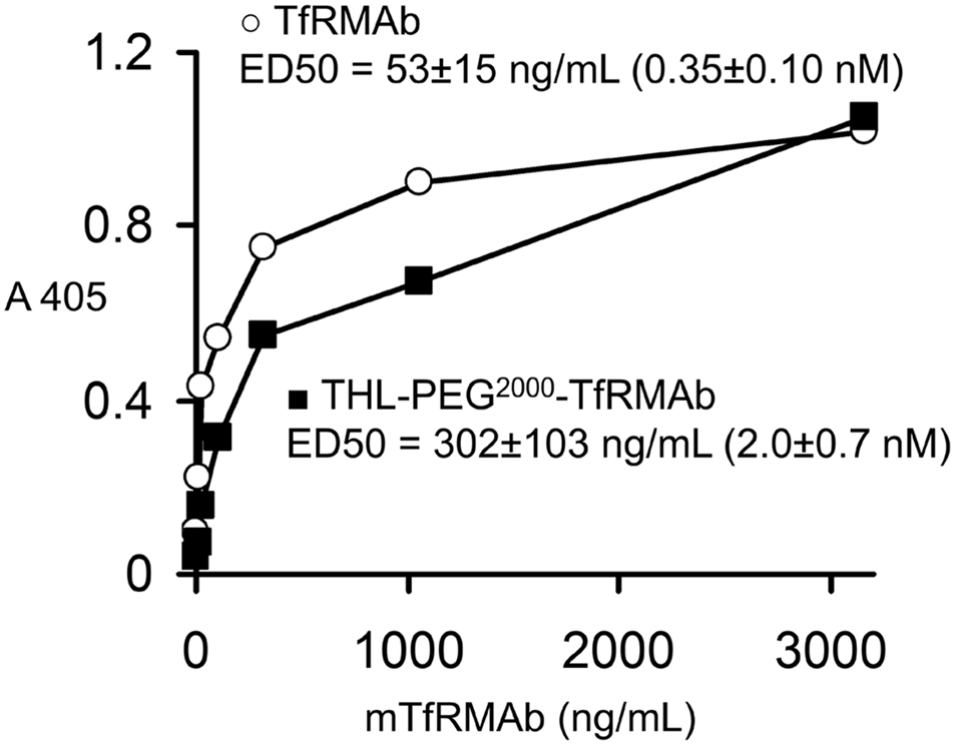
ELISA results of binding of either unconjugated recombinant TfRMAb or the DSPE-PEG^2000^-TfRMAb conjugate. The latter is formed following solubilization of TfRMAb-conjugated THLs with 0.2% Triton X-100. The thiolated TfRMAb forms a thio-ether linkage at the maleimide tip of the DSPE-PEG2000 phospholipid. The ED50 of binding was determined by non-linear regression analysis of the binding data (“Methods” section), and converted to nM based on the 150,000 Da molecular weight of the TfRMAb.

Lipofection of COS cells with the pPDGFB-NPC1 plasmid DNA resulted in a level of expression of the 180–200 kDa NPC1 protein comparable to the expression produced with the pCMV-NPC1 plasmid DNA (Fig. 1). A faint 200 kDa band is observed in the control cells and may represent endogenous COS cell NPC1. The bioactivity of the THL binding to the mouse TfR was confirmed by ELISA using the mouse TfR1 extracellular domain (ECD) as the capture agent (Fig. 2). The ED50 of binding of the unconjugated TfRMAb was 0.35 ± 0.10 nM and the binding of the TfRMAb conjugated to DSPE-PEG2000 via the thio-ether linkage had an ED50 of 2.0 ± 0.7 nM (Fig. 2). The TfRMAb targeted THL encapsulating the pGL4 luciferase expression plasmid produced high levels of luciferase gene expression following the application of freshly prepared THLs to mouse 3T3 cells (Table 1). Conversely, if the TfRMAb was replaced by rat IgG, then no luciferase gene expression was observed in the cells (Table 1). Incubation of the TfRMAb-THLs at 4 °C for either 1 or 5 days had no effect on luciferase gene expression (Table 1), which indicates the THLs are stable for up to 5 days when stored at 4 °C. TfRMAb-THLs encapsulated with the pPDGFB-NPC1 plasmid DNA showed no leakage of encapsulated plasmid DNA after storage at 4 °C for 4 days, based on either agarose gel electrophoresis or the Quantifluor DNA assay (“Methods” section).

**Table 1:** Luciferase expression (pg/mg protein) in mouse 3T3 fibroblasts at 24 or 48 h after application of THLs encapsulating the pGL4 luciferase expression plasmid DNA and targeted with either the TfRMAb or the isotype rat IgG.

THL storage at 4 °C (days)	TfRMAb/pGL4 THLs		Rat IgG/pGL4 THLs	
	24 h	48 h	24 h	48 h
0	55 ± 3	92 ± 9	< 1	< 1
1	66 ± 2	108 ± 7	< 1	< 1
5	62 ± 6	119 ± 2	< 1	< 1

Prior to application to the 3T3 cells, the THLs were stored at 4 °C for 0, 1, or 5 days. Data are mean ± SD (3 dishes per time point).

### NPC1^−/−^ mouse treatment study

Weekly IV treatment of the NPC1^m1N^ mice with either vehicle or TfRMAb targeted THLs encapsulating the pPDGFB-NPC1 plasmid was started when the mice were 44 days old, and mice were dosed a total of 5 consecutive weeks until the mice were so ill that euthanasia was necessary. There was no difference in survival between the treatment groups, and the female mice were euthanized at 77 ± 1 and 73 ± 5 days, respectively, following treatment with vehicle or THLs, and the male mice were euthanized at 75 ± 2 and 75 ± 3 days, respectively, following treatment with vehicle or THLs. At euthanasia, all mice demonstrated tremors, ruffled fur, yellow coat, and abnormal gait. There was no difference in body weight (BW) between the treatment groups at the end of the study (Table 2). The female mice had a BW of 17 ± 2 g and 18 ± 2 g at the start of the study in the vehicle and THL groups, respectively. The male mice had a BW of 23 ± 2 g and 23 ± 1 g at the start of the study in the vehicle group and THL groups, respectively. There was no statistical change in brain weight in the vehicle or THL treatment groups for either sex (Table 2). However, there was a significant 42% and 37% increase in spleen weight in the THL group as compared to the vehicle group in the females and males, respectively (Table 2). There was a significant 10% increase in liver weight in the male mice treated with THLs (Table 2). When the spleen and liver weights were normalized by BW for each mouse, the increase in spleen weight was 37% and 33% in the female and male groups, respectively (*P* < 0.001).

**Table 2:** Terminal organ weights (mg wet tissue) and body weights (grams) in female and male NPC1^−/−^ mice treated with either vehicle or THLs.

Sex	Treatment	Spleen	Liver	Brain	Body weight
Female	Vehicle	74 ± 18	969 ± 129	340 ± 39	13.3 ± 1.3
Male	Vehicle	86 ± 15	1,100 ± 102	360 ± 39	16.9 ± 1.0
Female	THL	105 ± 17^a^	994 ± 80	360 ± 19	13.9 ± 1.1
Male	THL	118 ± 14^b^	1,211 ± 100^c^	361 ± 28	17.5 ± 0.6

Mean ± SD (N = 8 female or 10 male mice per vehicle or THL treatment group).

^a^*P* < 0.005 by unpaired 2-tailed *T* test;

^b^*P* < 0.0001 by unpaired 2-tailed *T* test;

^c^*P* < 0.05 by Mann Whitney exact 2-tailed test.

**Figure 3:**
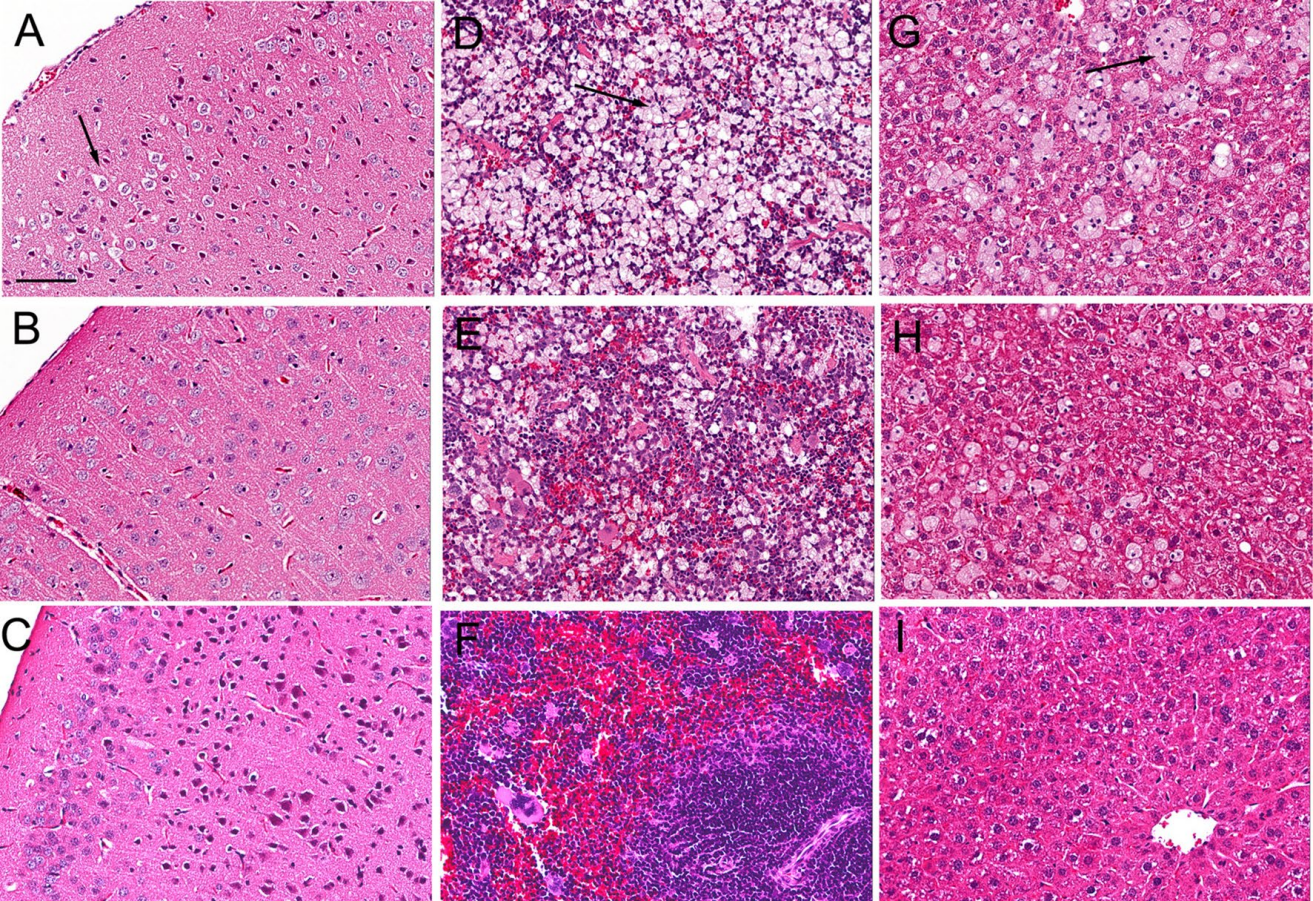
H&E stains of cerebral cortex (**A**–**C**), spleen (**D**–**F**), and liver (**G**–**I**) from NPC1^−/−^ mice or control mice at euthanasia. Panels (**A**, **D**, **G**) are NPC1^−/−^ mice treated with vehicle; panels (**B**, **E**, **H**) are NPC^−/−^ mice treated with TfRMAb/pPDGFB-NPC1 THLs. Panels (**C**, **F**, **I**) are control mice. Arrows in panels (**A**, **D**, **G**) point to cholesterol-laden cells in brain, spleen, and liver, respectively. Brain sections are through cortical layers I–IV. Magnification is the same for all panels and the panel A magnification bar = 100 microns.

**Figure 4:**
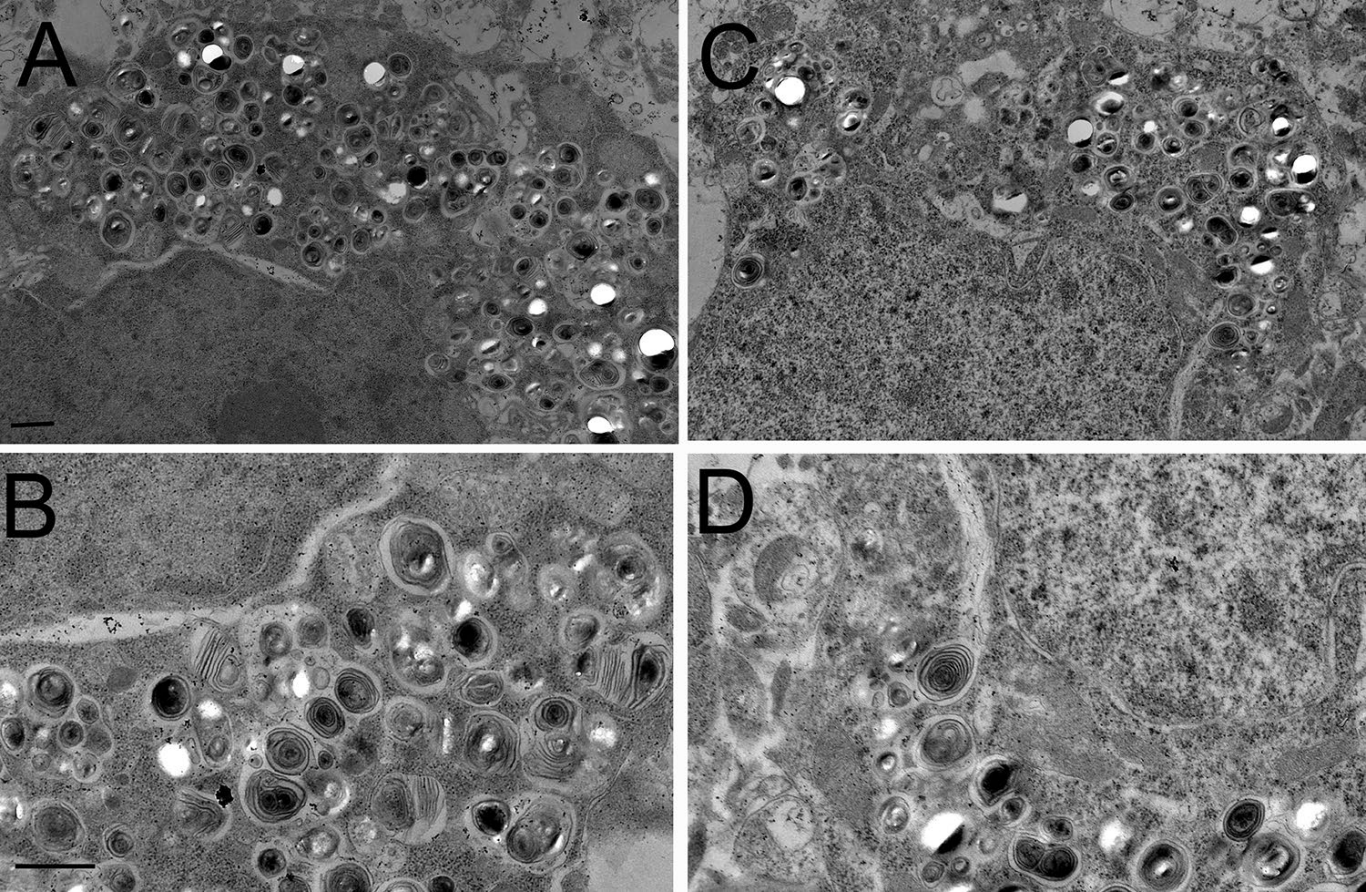
Electron microscopy of cerebral cortex of vehicle (**A**, **B**) or THL (**C**, **D**) treated NPC1^−/−^ mouse at euthanasia. Multi-lamellated vacuoles of varying size are present in the cytoplasm of brain cells. Magnification in panels (**A**) and (**C**) is the same and magnification bar in panel (**A**) is 1,000 nm; magnification in panels (**B**) and D is the same and magnification bar in panel (**B**) is 1,000 nm.

**Figure 5:**
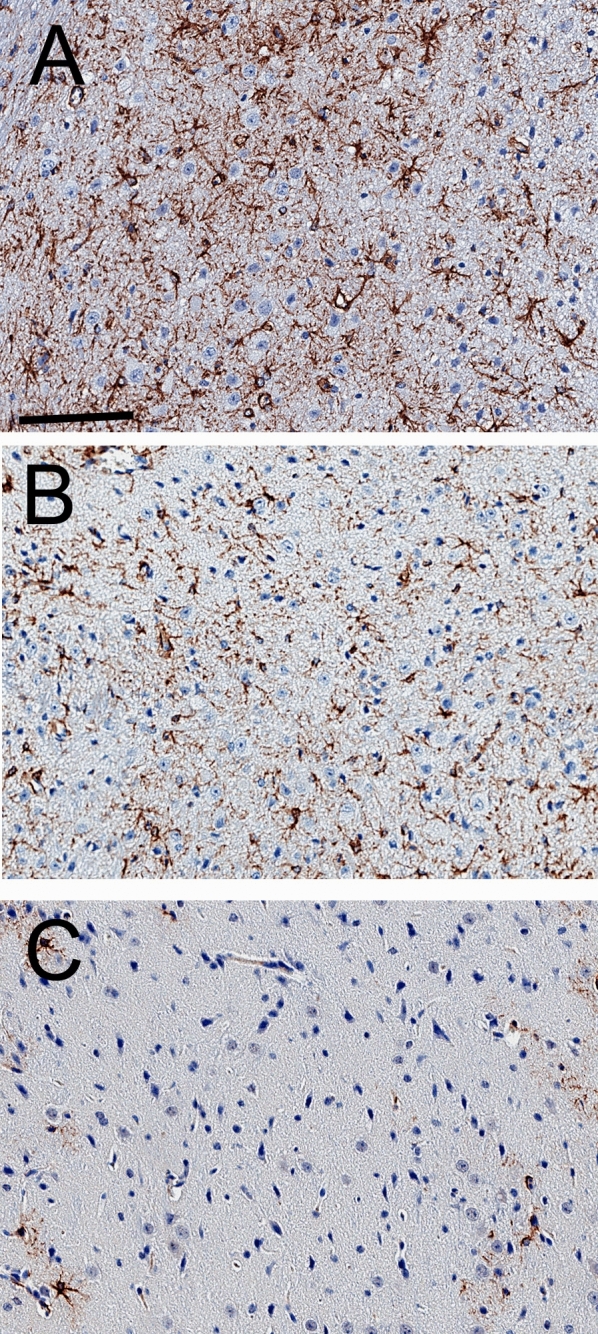
GFAP immunocytochemistry of thalamus of NPC1^−/−^ mouse treated with vehicle (**A**) or the TfRMAb/pPDGFB-NPC1 THLs (**B**). The GFAP immunocytochemistry of thalamus from a control mouse is shown in (**C**). Sections are counter-stained with hematoxylin. Magnification of all 3 panels is the same and the panel (**A**) magnification bar = 100 microns.

Brain hematoxylin and eosin (H&E) stains showed visible vacuolation of brain cells in the vehicle treated mice with no sex differences and a representative scan is shown in Fig. 3A, where an arrow marks a site of vacuolation. Electron microscopy of the terminal brain of the NPC1^−/−^ mouse, treated with either vehicle (Fig. 4A,B) or THLs (Fig. 4C,D) showed multiple vacuolations in the cytoplasm of brain cells and these vacuoles were frequently multi-lamellated. The vacuoles in brain were visibly reduced on the H&E stain in the THL treated mice (Fig. 3B), and no vacuolation was observed in brain of control mice (Fig. 3C). Glial fibrillary acidic protein (GFAP) immunohistochemistry showed extensive astrogliosis in the thalamus of the vehicle treated mice (Fig. 5A), and this astrogliosis was reduced in the THL treated mice (Fig. 5B), with minimal astrogliosis in the same region of the control mouse (Fig. 5C). The percent area of each section immune stained for GFAP was 12.9 ± 1.4% and 6.7 ± 0.8 (mean ± SD, N = 10 mice per group) for the vehicle and THL groups of mice, respectively (*P* < 0.0001). The astrogliosis in the cerebellum was even greater than that shown for the thalamus, and the cerebellar astrogliosis was not reduced by THL treatment (data not shown). H&E stains of the spleens of the vehicle treated mice showed extensive vacuolation of spleen cells (arrow, Fig. 3D) with no sex differences, and this vacuolation was reduced in the THL treated mice (Fig. 3E), with no vacuolation visible in the spleen of control mice (Fig. 3F). H&E stains of the livers of the vehicle treated mice showed extensive vacuolation of hepatic cells (arrow, Fig. 3G) with no sex differences, and this vacuolation was reduced in the THL treated mice (Fig. 3H), with no vacuolation visible in liver cells of control mice (Fig. 3I).

**Figure 6:**
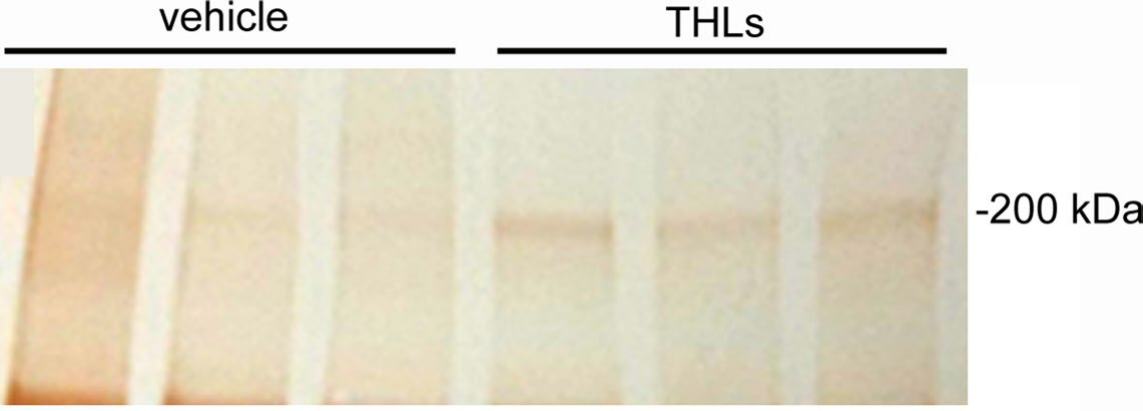
NPC1 Western blot of the spleen of 3 vehicle treated NPC^−/−^ mice and 3 THL treated NPC^−/−^ mice. The spleens of the THL treated mice express the 200 kDa immunoreactive NPC1 protein.

Quantitative PCR on organs removed 4 days following the last dose of vehicle or THLs showed high levels of pPDGFB-NPC1 plasmid DNA in target organs, and the plasmid abundance had a tissue rank order of spleen > liver > brain in the THL treated group (Table 3). The RT-PCR measurements of NPC1 mRNA in these organs is shown in Table 4. The Cq values for NPC1 were compared to the Cq values for mouse glyceraldehyde 3′-phosphate dehydrogenase (GAPDH). Treatment of the mice with THLs increased tissue NPC1 mRNA levels, as compared to the vehicle treated mice, whereas THL treatment caused no change in the Cq values for GAPDH. THL treatment caused an increase in the NPC1-GAPDH ΔCq values in a pattern, spleen > liver ≈ brain (Table 4), which paralleled the organ concentrations of the pPDGFB-NPC1 plasmid DNA (Table 3). The ΔΔCq values between THL and vehicle treatment groups showed the NPC1 mRNA, relative to the GAPDH mRNA, was increased 8,192-fold, 338-fold, and 238-fold in spleen, brain, and liver, respectively. NPC1 Western blot of spleen extracts showed detectable 200 kDa NPC1 protein in the THL treated mice, with no visible NPC1 protein in the vehicle treated mice (Fig. 6). The NPC1 protein paralleled the NPC1 mRNA, as immunoreactive NPC1 protein could not be detected with Western blot of extracts of brain or liver in the THL treated mice.

**Table 3:** Organ concentrations (pg plasmid DNA per mg wet tissue) in female and male NPC1^−/−^ mice at 4 days following the last IV injection of either vehicle or THLs.

Sex	Treatment	Brain	Spleen	Liver
Female	Vehicle	< 0.6	< 0.6	< 0.6
Male	Vehicle	< 0.6	< 0.6	< 0.6
Female	THL	10.1 ± 3.1	107.3 ± 40.5	39.9 ± 7.9
Male	THL	8.7 ± 0.6	58.6 ± 12.9	30.0 ± 7.8

Mean ± SEM (N = 4 male or 4 female mice per vehicle or THL treatment group). PCR Cq values were converted to pg plasmid DNA with a pPDGF-NPC1 plasmid DNA standard curve.

**Table 4:** Organ enrichment of NPC1 mRNA, relative to GAPDH mRNA, in THL treated NPC1^−/−^ mice as compared to vehicle treated NPC1^−/−^ mice.

Organ	Treatment	NPC1 Cq	GAPDH Cq	ΔCq	ΔΔCq	NPC1 mRNA fold change
Spleen	Vehicle	35.5 ± 2.6	18.8 ± 1.1	16.9 ± 2.1	–	–
Spleen	THL	22.8 ± 0.7	18.9 ± 0.4	3.9 ± 1.0	13.0	8,192
Liver	Vehicle	33.9 ± 2.8	24.2 ± 1.7	10.0 ± 2.6	–	–
Liver	THL	26.4 ± 0.9	24.3 ± 1.3	2.1 ± 1.0	7.9	238
Brain	Vehicle	37.0 ± 1.4	21.4 ± 0.4	15.9 ± 1.2	–	–
Brain	THL	29.6 ± 0.8	22.1 ± 1.6	7.5 ± 1.8	8.4	338

ΔCq = (NPC1 Cq − GAPDH Cq); ΔΔCq = [ΔCq (vehicle mice) − ΔCq (THL mice)]; NPC1 mRNA fold change between vehicle mice and THL mice computed from base 2 anti-log. Cq values are mean ± SD [8 mice (4 males; 4 females)] in vehicle group; 8 mice [(4 males; 4 females)] in THL group.

Chronic treatment of the NPC1^−/−^ mice with THLs caused the formation of a low titer anti-drug antibody (ADA) response against the TfRMAb protein. The OD/μL was 0.16 ± 0.08, in the vehicle treated group with no sex differences, whereas the OD/μL was 1.57 ± 0.02 in the THL treated group with no sex differences. These ADA titers are low compared to a significant ADA response, which can produce ADA titers ≫ 100 OD/μL^19^.

## Discussion

This investigation attempts to treat the NPC1^m1N^ null mouse with intravenous plasmid DNA therapy using THLs that target the mouse TfR1, and an expression plasmid DNA, designated pPDGFB-NPC1, encoding the 3.9 kb human NPC1 open reading frame driven by a 1.5 kb promoter taken from the human PDGFB 5′-flanking sequence. Weekly IV injections of THLs or vehicle was administered to a cohort of 36 NPC1^−/−^ mice starting at the age of 44 days (6–7 weeks) at a dose of 6 μg plasmid DNA per mouse. The findings of this study are consistent with the following conclusions. First, weekly IV administration of THLs produces high levels of the pPDGFB-NPC1 plasmid DNA in the target organs (brain, spleen, liver) as shown by quantitative PCR (Table 3), and that the plasmid is transcribed to produce tissue levels of the NPC1 mRNA in proportion to the tissue plasmid DNA level (Tables 3, 4). Second, chronic THL administration produces visible reductions in the vacuolation of cells in liver and spleen (Fig. 3), which correlates with increases in weights of these organs, particularly the spleen (Table 2). This finding parallels the observations of other studies of the NPC1^−/−^ mouse, which showed the spleen weight is increased in the untreated NPC1^−/−^, relative to control mice, and that spleen weights are increased further by therapy^20^. Third, chronic THL administration produces visible reductions in the vacuolation of cells in the cerebral cortex (Fig. 3A,B), which correlates with a significant reduction in astrogliosis, based on quantitation of GFAP immunocytochemistry (“Results” section and Fig. 5). Fourth, initiation of THL therapy at 6–7 weeks of age is too late to reverse the severe clinical effects of congenital NPC1 absence, and THL therapy caused no change in the decline in body weight or early death at 10–11 weeks (“Results” section).

The NPC1^−/−^ mouse replicates many of the findings of human NPC1, including cholesterol storage disease in brain, spleen, and liver, neurodegeneration associated with astrogliosis and severe vacuolation of brain cells^2,3^. The disease in the NPC1^−/−^ mouse is eliminated by insertion of the 3.9 kb murine NPC1 cDNA under the influence of a 3.6 kb prion promoter^21^. Therefore, gene therapy offers a promising approach to the treatment of NPC1, which currently has no FDA approved therapy. The standard approach to gene therapy of the brain is the use of adeno-associated virus (AAV), and certain AAV serotypes, such as AAV9, cross the BBB following IV administration^8^. The use of ssAAV, which has twice the cloning capacity of scAAV^9^, and the development of mini-promoters, allows for the insertion of a large therapeutic gene such as the 3.9 kb NPC1 open reading frame into the AAV genome^22,23,24^. The NPC1 gene with a 227 nucleotide elongation factor-1α (EF1α) promoter was delivered to NPC1^−/−^ mouse brain with AAV9 following IV administration of high injection doses (ID), 1–2 × 10^14^ vg/kg of the virus^22^. Treatment caused an increase in lifespan from 69 ± 3 days to 166 ± 89 days, and 20–30% of brain cells were transduced in the regions studied, layer V of the cortex and the CA3 region of the hippocampus^22^. In a second study of treatment of the NPC1^−/−^ mouse with the NPC1 gene encoded in AAV9 under the influence of the CMV promoter, the virus was administered by injection into the left cardiac ventricle at a high ID of 1 × 10^14^ vg/kg in 4 day old mice, which resulted in a modest increase in lifespan from 71 ± 4 days to 94 ± 4 day^23^. In a third study, a low dose, 5 × 10^12^ vg/kg, or a high dose, 2 × 10^14^ vg/kg, of AAV9 carrying the NPC1 gene, under the influence of the human synapsin 1 promoter, was administered into both lateral ventricles of a newborn mouse^24^. This bilateral intra-cerebroventricular (ICV) route of administration of the AAV9 bypassed the BBB. The low ID produced an increase in lifespan no greater than that produced by daily oral high dose (1,200 mg/kg/day) miglustat, whereas the high ID increased lifespan to an average of 158 days^24^. The longer lifespan after bilateral ICV injection of high doses of AAV9 is attributed to the greater distribution of the AAV9 within the brain following a bilateral ICV injection in the newborn mouse. Prior work shows widespread gene distribution in the brain following ICV administration in the newborn mouse^25^, which is attributed to the shorter diffusion distances in the small brain of a mouse^7^, particularly the brain of a 1 g newborn mouse. No matter the route of administration, the effect of NPC1 gene therapy in the NPC1^−/−^ mouse requires a high ID of 10^14^ vg/kg^22,23,24^. However, this ID of 10^14^ vg/kg of AAV may be genotoxic, as a 10^14^ vg/kg dose of AAV administered as a single injection in newborn mice produces a 70% incidence of hepatic carcinoma in mice observed for up to 21 months^26^. AAV-induced liver cancer was dependent on the promoter/enhancer used in the vector design^26^, and liver cancer has not been observed in humans. Nevertheless, the theoretical concerns of AAV genotoxicity provides the rationale for parallel examination of plasmid DNA-based gene therapy. Plasmid DNA gene therapy requires a DNA drug delivery system, and the THL delivery technology was developed for this purpose^11^.

Plasmid DNA as large as 22 kb has been successfully encapsulated in THLs, followed by expression in brain after IV administration^14^. Therefore, there was no restriction on the size of the promoter that could be used in the engineering of a NPC1 expression plasmid. The 1.5 kb human PDGFB promoter^15^ was used, which produced a 6.0 kb expression cassette, and a 8.0 kb plasmid DNA (“Methods” section). The PDGFB promoter is expressed in neurons, and produces a greater level of transgene expression in brain, as compared to the CMV promoter^18^. The pPDGFB-NPC1 plasmid DNA was delivered to brain, spleen, and liver with intravenous THLs, and the tissue plasmid DNA concentrations at 4 days following the last IV dose of the study are shown in Table 3. The brain plasmid DNA concentration is 10 pg DNA per mg tissue (Table 3), which corresponds to 1.1 × 10^6^ DNA molecules per mg brain tissue, based on the molecular weight of a 8.0 kb double stranded plasmid DNA of 5.3 × 10^6^ g/mole. There are 100 million cells per 400 mg mouse brain^27^, or 0.25 × 10^6^ cells/mg brain. These calculations indicate there are 4–5 plasmid DNA molecules delivered per each cell in brain of the mouse with the THL delivery system, which correlates with prior estimates of THL delivery of plasmid DNA to brain cells in the primate^28^. There is an even greater number of plasmid DNA molecules delivered to spleen and liver cells, with spleen > liver (Table 3). The greater uptake of THLs by spleen as compared to liver is attributed to the much higher expression of the TfR1 isoform in spleen as compared to liver^29^, as it is the TfR1 isoform that is targeted by the TfRMAb used in this study^30^.

The pPDGFB-NPC1 plasmid DNA is transcribed in the target organs as RT-PCR shows an enrichment of the human NPC1 mRNA (Table 4), relative to GAPDH mRNA, in the organs in proportion to the tissue level of plasmid DNA (Table 3). This expression of the NPC1 gene in the target organs correlates with a visible reduction in the number of vacuolated cells in brain, liver, or spleen of the THL treated mice, as compared to the vehicle treated mice (Fig. 3), and to a reduction in cerebral cortical astrogliosis in the THL treated mice (Fig. 5). However, THL therapy, which started at day 44 (6–7 weeks) did not improve either weight loss or survival in the NPC1^−/−^mice (“Results” section).

The lack of a therapeutic effect on survival in the NPC1^−/−^ mouse, when therapy is not initiated until 6–7 weeks, suggests disease stage at the start of therapy determines the therapeutic outcome, as described previously for other lysosomal storage disorders that affect the brain^31^. The severe accumulation in brain of autophagic lysosomal inclusion bodies, such as those found at termination in this study (Fig. 4), are present in the brain of the NPC1^−/−^ mouse as early as 6 weeks of age, or when therapy was initiated in this study^32^. The astrogliosis observed in this study (Fig. 5), as well as microglial activation, begins in brain of the NPC1^−/−^ mouse as early as 2 weeks of age^33,34^. Suppression of myelin production begins as early as 5 weeks of age^35^.

In conclusion, this investigation shows that the NPC1 plasmid DNA can be delivered to target organs of the NPC1 mouse with intravenous administration of THLs that target the TfR, but that therapy must be initiated much earlier than 6–7 weeks of age in the NPC^−/−^ mouse. Since TfRMAb targeted THLs cross the placenta^36^, plasmid DNA therapy of the NPC1^−/−^ mouse could even begin in utero.

## Methods

### Plasmid DNA

A luciferase expression plasmid, pGL4.13, and designated pGL4, was purchased from Promega (Madison, WI). A human NPC1 expression plasmid (#SC120010), under the influence of the CMV promoter, is designated pCMV-NPC1, and was purchased from Origene (Rockville, MD). A human NPC1 expression plasmid, under the influence of the human PDGF-B promoter, was custom synthesized as a 5.6 kb expression cassette, which was subcloned in a 8.0 kb pUC plasmid DNA at Genscript (Piscataway, NJ), and the sequence of the entire expression cassette was confirmed by bidirectional DNA sequencing. The NPC1 open reading frame corresponded to nucleotides 164–4,000 of NM_000271, and was preceded on the 5′ end by a 9 nucleotide (nt) Kozak sequence, and a 1.5 kb human PDGF-B promoter, and on the 3′ end by the bovine growth hormone (BGH) poly A sequence, which corresponded to nt 2,974–3,198 of MK988448. The sequence of the PDGF-B promoter corresponds to nt − 1,360 to + 75, relative to the transcription start site^15^. The transcription start site was identified by Blast alignment of the human PDGF-B mRNA, NM_002608, and the human PDGF-B gene, NG_002599. The PDGFB-NPC1-BGH expression cassette were confirmed by bidirectional DNA sequencing, and encoded for the human NPC1 protein corresponding to amino acids (AA) 1–1,278 of the human NPC1 protein sequence, NP_000262. Transfection grade (low endotoxin) gigapreps of all plasmids were prepared, and plasmid constructs were confirmed by restriction endonuclease digestion and agarose gel electrophoresis.

### Production of recombinant TfRMAb

THL delivery to the mouse brain targets the mouse TfR type 1 with the 8D3 rat MAb against the mouse TfR, which was previously produced from hybridoma generated ascites^37^. The AA sequences of the variable region of the heavy chain, VH, and the variable region of the light chain, VL, of this antibody has been described previously^30^. A recombinant form of the 8D3 TfRMAb was produced following the engineering of separate heavy chain (HC) and light chain (LC) expression cassettes. The HC expression plasmid encoded for the 118 AA 8D3 VH followed by the 322 AA rat IgG2a constant region, and the LC expression plasmid encoded for the 108 AA 8D3 VL and the 106 AA rat kappa B allele constant region. HC and LC synthetic genes were produced at Genscript (Piscataway, NJ), and subcloned, with a signal peptide in a eukaryotic expression plasmid with a CMV promoter and a human growth hormone polyA sequence. The expression plasmids were confirmed by bidirectional DNA sequencing. Chinese hamster ovary (CHO) K1 cells were dual electroporated, followed by propagation of the cells in serum free medium in a 14 day fed batch culture, and the TfRMAb was affinity purified with protein G to yield 3 g of recombinant TfRMAb. The purity, identity, safety, and potency of the TfRMAb was confirmed by reducing and non-reducing sodium dodecylsulfate polyacrylamide gel electrophoresis (SDS-PAGE), size exclusion HPLC, mouse IgG Western blotting, and a TfR binding ELISA with the mouse TfR1 ECD (VWR, Radnor, PA). The endotoxin content of the TfRMAb was low, < 0.5 EU/mg, as determined with the Limulus amebocyte lysate (LAL) spectrophotometric assay (ThermoFisher Scientific, Waltham, MA).

### Lipofection and NPC1 Western blotting

COS cells were obtained from the American Type Culture Collection (ATCC, Manassas, VA), and grown in Eagle medium with 10% fetal bovine serum. Cells were plated in 6-well dishes and lipofected with 2 μg/dish of either pCMV-NPC1 or pPDGFB-NPC1 and Lipofectamine 2000 (ThermoFisher, Carlsbad, CA). After 2 days of transfection, washed monolayers were lysed and separated by reducing SDS-PAGE followed by blotting to nitrocellulose with an iBlot2 (ThermoFisher). The filters were probed with a 1:1,000 dilution of a rabbit polyclonal antibody directed against the C-terminus of human NPC1 (NB400-148, Novus Biologicals, Littleton, CO), a sheep anti-rabbit secondary antibody from Bethyl Labs (Montgomery, TX), and the ABC Vectastain reagent (Vector Labs, Burlingame, CA), followed by color development with diaminobenzidine. High molecular weight standards were run in parallel.

### THL production

Phospholipids were purchased from Avanti Polar Lipids (Alabaster, AL), and included 1-palmitoyl-2-oleoyl-*sn*-glycerol-3-phosphocholine (POPC), dimethyldioctadecylammonium bromide (DDAB), distearoyl-sn-phosphoethanolamine-N-[maleimide(polyethyleneglyco)-2000] (DSPE-PEG^2000^-MAL), 1,2-distearoyl-*sn*-glycero-3-phosphoethanolamine-N-[methoxy(polyethylene glycol)-2000] (DSPE-PEG^2000^), and ovine wool cholesterol. THLs were prepared as described previously^11^, with 3 modifications. First, the amount of phospholipid used to encapsulate 400 μg DNA was reduced fourfold from 40 to 10 μmol, as this was found to reduce the number of DNA-free or empty liposomes to < 10% of total. Second, cholesterol was added at 6 mol% to the phospholipid to enable storage of the THLs at 4C for several days prior to administration to NPC1 mice. This level of cholesterol stabilizes membranes^38^. Third, the plasmid DNA encapsulation in the liposomes is enhanced with the addition of ethanol^39,40^. A 10 μmol mixture of phospholipid [8.7 μmol POPC, 0.6 μmol cholesterol, 0.3 μmol DDAB, 0.3 μmol DSPE-PEG^2000^, 0.1 μmol DSPE-PEG^2000^-maleimide (MAL)] dissolved in chloroform was evaporated to a thin film, and phospholipids were hydrated by vortexing and brief bath sonication in 400 μL of 0.05 M 4-(2-hydroxyethyl)-1-piperazineethanesulfonic acid buffered water (HBW, pH = 7.0), followed by the addition of 400 μg plasmid DNA in Tris–EDTA (TE) buffer. An equal volume of 80% ethanol was added. Following a series of freeze/thaw cycles, the phospholipid/DNA mix was extruded several times through an LF-1 extruder (Avestin, Ottawa, Canada) with a 200 nm polycarbonate filter. As the ethanol inhibits nuclease activity^40^, the ethanol was removed by dialysis using a Slide-A-Lyzer with a 20 kDa MW cut-off (ThermoFisher). The THL preps were produced in duplicate, which were pooled prior to dialysis, and non-encapsulated plasmid DNA was removed by treatment with bovine pancreatic DNase I (Sigma, St. Louis, MO) and Ecoli exonuclease II (ThermoFisher)^11^. Prior to conjugation of the TfRMAb to the maleimide (MAL) moieties on the surface of the liposome, the MAb (2.5 mg) was thiolated with a 15:1 molar ratio of 2-iminothiolane (Sigma), which produced 2–3 thiol groups per MAb, based on the reaction with Ellman’s reagent (Sigma). The unconjugated MAb and the nuclease digested DNA was removed by Sepharose CL-4B gel filtration chromatography in 0.05 M Hepes buffer (pH = 7.0)^11^, and the THL peak in the void volume was pooled and micro-filtered with a 0.2 micron Millex GV filter unit (Millipore-Sigma), and stored at 4 °C until either analysis or overnight shipping on cold packs to The Jackson Laboratory (JAX, Bar Harbor, ME). The typical THL prep had DNA concentration of 40 μg/mL, a TfRMAb concentration of 100 μg/mL, and a phospholipid concentration of 1.5 mg/mL. Approximately 40–50 TfRMAb molecules were conjugated to each liposome, and the number of non-encapsulated or “empty” liposomes was < 10%. The diameter of the THL was 100–150 nm as determined by a dynamic light scattering particle analyzer (Nicomp model N3000, Entegris, Billerica, MA). Following agarose gel electrophoresis and ethidium bromide staining, all of the plasmid DNA was trapped at the top of the gel, which indicates the DNA is fully encapsulated. Following solubilization of the THLs with 0.2% Triton X-100, the plasmid DNA entered the gel and showed the same migration as the untreated super-coiled DNA. The retention of the plasmid DNA in the interior of the THL was confirmed with the QuantiFluor dsDNA System (Promega) using a Quantus fluorometer (Promega). The endotoxin content was measured with the LAL assay and was 0.06–0.12 endotoxin units (EU)/mL.

### TfRMAb ELISA

The conjugation of the TfRMAb to the THL results in the formation of DSPE-PEG^2000^-S-TfRMAb within the intact THL, where the -S- represents the thioether bond conjugating the TfRMAb to the maleimide (MAL) moiety at the tip of the PEG^2000^ group on the surface of the THL. This lipid-PEG^2000^-S-MAb unit is released from the THL following solubilization with 0.2% Triton X-100. The binding of the THL-PEG-TfRMAb to the mouse TfR1 ECD was compared to the unconjugated TfRMAb with an ELISA, where the capture agent was the mouse TfR1 ECD, and the detector reagent was a conjugate of alkaline phosphatase and a goat anti-rat kappa light chain antibody (Millipore-Sigma) with measurement of colorimetric absorption (Abs) at 405 nm. Binding of either the unconjugated TfRMAb, or the THL-PEG-TfRMAb, was measured between 3 and 3,000 ng/mL using an iMark microplate absorbance reader (BioRad). Rat IgG alone produced no ELISA signal. The concentration of antibody that produces 50% of maximal binding, ED50, was determined by non-linear regression by fitting the data to: Abs = (Amax·S)/(EC50 + S), where Amax is the maximal Abs and S = the concentration of TfRMAb. The EC50 was estimated with the units of ng/mL, and converted to units of nM, based on a MAb MW of 150,000 Da. The same MW was used for either the unconjugated TfRMAb or the DSPE-PEG^2000^-MAL-TfRMAb, because the amount of the latter added to the ELISA wells was based on protein content as determined with the bicinchoninic acid (BCA) protein assay (ThermoFisher).

### Luciferase gene expression

The stability of the THLs with storage at 4 °C was determined by encapsulation of the pGL4 luciferase expression plasmid DNA in TfRMAb targeted THLs. The THLs were stored at 4C for up to 5 days prior to application (2 μg plasmid DNA per 35 mm dish) to cultured mouse 3T3 fibroblasts (ATCC), and intracellular luciferase enzyme activity was determined with the Bright-Glo Luciferase assay (Promega) and a Glo-Max 20/20 luminometer (Promega). The relative light units (RLU) were converted to pg luciferase with a standard curve using recombinant firefly luciferase (Promega), and the data normalized per mg protein in the well based on the BCA assay.

### Npc1^−/−^ mouse treatment study

A colony of 36 Npc1^−/−^ mice, also called the NPC1^*m1N*^ mouse (16 females, 20 males), was developed at The Jackson Lab (JAX, Bar Harbor, ME) for this study (JAX stock #003092).The study protocol was approved by the JAX Institutional Animal Care and Use Committee, and all experiments were performed in accordance with relevant guidelines and regulations. Genotype of all mice was confirmed by tail PCR. The mice were divided into 2 treatment groups of 18 mice (8 females, 10 males), with the first group treated by vehicle (Hepes buffer, pH = 7.0), and the second group treated with TfRMAb-targeted THLs encapsulating the pPDGFB-NPC1 plasmid DNA (designated TLC-200). Each mouse was treated by weekly tail vein injections of a volume of 150 μL. The injection dose (ID) in the THL treated mice was 6 μg plasmid DNA per mouse, and the ID of the TfRMAb was 15 μg per mouse. THLs were not frozen prior to injection, and it was necessary for JAX to perform sterility testing on each batch. THLs were shipped on cold packs on Monday of each week, received by JAX on Tuesday, with sterility results available on Thursday, and IV injections performed on Friday, or 4 days following THL production. Weekly IV treatment began at 44 days (6–7 weeks) of age. Mice were monitored weekly with body weights and clinical signs (tremor, ruffled fur, yellow coat, abnormal gait). At euthanasia, brain, liver, and spleen were removed from all mice for organ weights. The organs were then divided in two parts, with the first part flash frozen on dry ice, and shipped to the sponsor overnight on dry ice. The second part was placed in cold 4% paraformaldehyde and shipped on cold packs overnight to the sponsor.

### Organ histology

The fixed tissues were embedded in paraffin for 5 micron sections followed by either H&E staining or GFAP immunohistochemistry at the UCLA Tissue Pathology Core Lab. Sections were performed on brains from 20 mice (10 from each treatment group), and on liver and spleen from 12 mice (6 from each treatment group). Digital images at 20X magnification were obtained for all 64 slides with an Aperio Image system (Leica Biosystems, Buffalo Grove, IL). The GFAP immune staining was quantified as percent area with NIH Image J (version 1.52a) for the thalamus of the vehicle and THL treated NPC1^−/−^ mice. Sections were quantified for 10 mice (5 male, 5 female) for the vehicle treated group, and for 10 mice (5 male, 5 female) for the THL treated group, and 3 fields per section were averaged. Statistical differences between the vehicle and THL treated mice were assessed with the unpaired Student’s *t* test. Transmission electron microscopy was performed on brains from 2 vehicle treated mice and 2 THL treated mice. The brain in paraformaldehyde fixative was enriched to 2% glutaraldehyde, treated with 1% OsO4 and embedded in epon. Ultra-thin (50 nm) sections on 200 mesh copper grids were stained with uranyl acetate and lead citrate and examined at 60 kV with a JEOL JEM-100CX II electron microscope at the UCLA Brain Research Institute core laboratory. Photographs were taken with an AMT XR611camera at 2.5 s exposure.

### Quantitative PCR and RT-PCR

The mice were euthanized at 4 days following the last weekly dose of vehicle or THL. The frozen brain, liver, and spleen (40 mg) was homogenized and plasmid DNA isolated with a QIAprep Spin Miniprep kit (Qiagen, Valencia, CA). The pPDGFB-NPC1 plasmid DNA per mg wet tissue weight was quantified by real time PCR using a CFX Connect Real-Time PCR Detect System (BioRad, Hercules, CA), specific PCR primers, and a standard curve with serial dilutions (1.7 × 10^3^ to 1.7 × 10^9^) plasmid copy number (PCN) per μL of the pPDGFB-NPC1 plasmid DNA. The Cq (earliest cycle of fluorescence detection) varied from 8.2 at 10^9^ PCN/μL to 29.3 at 10^3^ PCN/μL, and the Cq was > 36 when no plasmid DNA was added to the mix. The forward and reverse PCR primers were designed at the IDT Real Time qPCR Assay Entry website based on a sequence of the human NPC1 mRNA (NM_000271) with mismatches to the mouse NPC1 mRNA (AF003348). The forward (sense) primer sequence is 5′-CTCCCAGTATGTTCCTGTCATC-3′ and the reverse (antisense) primer sequence is 5′-AATCCCGCAAAGAGAGAGAAG-3′, which produces an amplicon length of 103 nucleotides, a Tm of 62 °C, and a GC content of 48–50%. PCR was performed with the PowerUp SYBR Green Master Mix (ThermoFisher) and 40 cycles of denaturation (95C, 15 s) and annealing (51.3C, 30 s). The Cq (earliest cycle of fluorescence detection) was determined for both the pPDGFB-NPC1 plasmid standard and the tissue samples, and tissue Cq was converted to pg pPDGFB-NPC1 plasmid based on the standard curve.

NCP1 mRNA was measured for brain, liver, and spleen by reverse transcriptase (RT)-PCR. The tissue Cq value for NPC1 was compared to the Cq value for a housekeeping gene, glyceraldehyde 3′-phosphate dehydrogenase (GAPDH). Although GAPDH transcripts may vary among tissues^41^, there was no difference in organ Cq values for GAPDH between the vehicle and THL treated mice (Table 4). The mouse GAPDH primers were derived from the mouse GAPDH mRNA sequence (XM_017321385). The forward (sense) primer sequence is 5′-GGGTGTGAACCACGAGAAATA-3′ and the reverse (antisense) primer sequence is 5′-GTCATGAGCCCTTCCACAAT-3′, which produces an amplicon length of 129 nucleotides, a Tm of 62 °C, and a GC content of 48–50%. Organ RNA was isolated from homogenates with the RNeasy Mini Kit (Qiagen), and the RNA was quantified with the QuantiFluor RNA System (Promega). cDNA was produced from the RNA with the iScript Reverse Transcription Supermix (Biorad). RT-PCR measurements were performed twice, and each sample was measured in triplicate in each assay. PCR was performed with both the mouse NPC1 and the mouse GAPDH primers. The ΔCq is the difference in Cq value for NPC1 and GAPDH for a given organ. The ΔΔCq is the difference in ΔCq for the vehicle treated mouse and the THL treated mouse. The change in NPC1 mRNA abundance in the organ of the THL treated mouse was computed from the base 2 antilog of the ΔΔCq using the Omni antilog calculator.

### Anti-drug antibody ELISA

The anti-drug antibody (ADA) response against the TfRMAb following chronic treatment of NPC1^−/−^ mice with either vehicle or THLs was measured on the terminal serum by ELISA. The capture agent for the ADA ELISA is the recombinant TfRMAb, and the detector agent in the ELISA is a complex of biotinylated TfRMAb and a conjugate of streptavidin and horseradish peroxidase (Vector Labs), and color development, at 490 nm, was measured with the ortho-phenylenediamine chromagen. The TfRMAb was biotinylated with sulfo-NHS-LC-LC-biotin (ThermoFisher), where NHS = N-hydroxysuccinimide, and LC = long chain. The biotinylation of the TfRMAb was confirmed by Western blotting with the ABC vectastain reagent (Vector Labs). The ADA titer was reported as the optical density (OD) per μL mouse serum^19^.

## References

[1] Burlina A (2014). Niemann-Pick disease type C: introduction and main clinical features. J. Neurol..

[2] Pentchev PG (1980). A lysosomal storage disorder in mice characterized by a dual deficiency of sphingomyelinase and glucocerebrosidase. Biochim. Biophys. Acta.

[3] Loftus SK (1997). Murine model of Niemann-Pick C disease: mutation in a cholesterol homeostasis gene. Science.

[4] Ramirez CM (2010). Weekly cyclodextrin administration normalizes cholesterol metabolism in nearly every organ of the Niemann-Pick type C1 mouse and markedly prolongs life. Pediatr. Res..

[5] Pontikis CC, Davidson CD, Walkley SU, Platt FM, Begley DJ (2013). Cyclodextrin alleviates neuronal storage of cholesterol in Niemann-Pick C disease without evidence of detectable blood-brain barrier permeability. J. Inherit. Metab. Dis..

[6] Ory DS (2017). Intrathecal 2-hydroxypropyl-beta-cyclodextrin decreases neurological disease progression in Niemann-Pick disease, type C1: a non-randomised, open-label, phase 1–2 trial. Lancet.

[7] Pardridge WM (2016). CSF, blood-brain barrier, and brain drug delivery. Expert. Opin. Drug. Deliv..

[8] Foust KD (2009). Intravascular AAV9 preferentially targets neonatal neurons and adult astrocytes. Nat. Biotechnol..

[9] Hudry E (2018). Efficient gene transfer to the central nervous system by single-stranded Anc80L65. Mol. Ther. Methods Clin. Dev..

[10] Gray SJ (2011). Preclinical differences of intravascular AAV9 delivery to neurons and glia: a comparative study of adult mice and nonhuman primates. Mol. Ther..

[11] Pardridge WM (2010). Preparation of Trojan horse liposomes (THLs) for gene transfer across the blood-brain barrier. Cold Spring Harb. Protoc..

[12] Zhang Y, Jeong Lee H, Boado RJ, Pardridge WM (2002). Receptor-mediated delivery of an antisense gene to human brain cancer cells. J. Gene Med..

[13] Zhang Y, Schlachetzki F, Pardridge WM (2003). Global non-viral gene transfer to the primate brain following intravenous administration. Mol. Ther..

[14] Xia CF (2007). Comparison of cDNA and genomic forms of tyrosine hydroxylase gene therapy of the brain with Trojan horse liposomes. J. Gene Med..

[15] Sasahara M (1991). PDGF B-chain in neurons of the central nervous system, posterior pituitary, and in a transgenic model. Cell.

[16] Fries JW, Collins T (1992). Platelet-derived growth factor expression in a transgenic model. Kidney Int..

[17] Rockenstein EM (1995). Levels and alternative splicing of amyloid beta protein precursor (APP) transcripts in brains of APP transgenic mice and humans with Alzheimer's disease. J. Biol. Chem..

[18] Paterna JC, Moccetti T, Mura A, Feldon J, Bueler H (2000). Influence of promoter and WHV post-transcriptional regulatory element on AAV-mediated transgene expression in the rat brain. Gene Ther..

[19] White JT (2008). Development, validation, and clinical implementation of an assay to measure total antibody response to naglazyme (galsulfase). AAPS J..

[20] Nesslauer AM (2019). A therapy with miglustat, 2-hydroxypropyl-ss-cyclodextrin and allopregnanolone restores splenic cholesterol homeostasis in Niemann-pick disease type C1. Lipids Health Dis..

[21] Loftus SK (2002). Rescue of neurodegeneration in Niemann-Pick C mice by a prion-promoter-driven Npc1 cDNA transgene. Hum. Mol. Genet..

[22] Chandler RJ (2017). Systemic AAV9 gene therapy improves the lifespan of mice with Niemann-Pick disease, type C1. Hum. Mol. Genet..

[23] Xie C, Gong XM, Luo J, Li BL, Song BL (2017). AAV9-NPC1 significantly ameliorates Purkinje cell death and behavioral abnormalities in mouse NPC disease. J. Lipid. Res..

[24] Hughes MP (2018). AAV9 intracerebroventricular gene therapy improves lifespan, locomotor function and pathology in a mouse model of Niemann-Pick type C1 disease. Hum. Mol. Genet..

[25] Passini MA, Wolfe JH (2001). Widespread gene delivery and structure-specific patterns of expression in the brain after intraventricular injections of neonatal mice with an adeno-associated virus vector. J. Virol..

[26] Chandler RJ (2015). Vector design influences hepatic genotoxicity after adeno-associated virus gene therapy. J. Clin. Invest..

[27] Williams RW (2000). Mapping genes that modulate mouse brain development: a quantitative genetic approach. Results Probl. Cell Differ..

[28] Chu C, Zhang Y, Boado RJ, Pardridge WM (2006). Decline in exogenous gene expression in primate brain following intravenous administration is due to plasmid degradation. Pharm. Res..

[29] Chua AC (2010). Iron uptake from plasma transferrin by a transferrin receptor 2 mutant mouse model of haemochromatosis. J. Hepatol..

[30] Boado RJ, Zhang Y, Wang Y, Pardridge WM (2009). Engineering and expression of a chimeric transferrin receptor monoclonal antibody for blood-brain barrier delivery in the mouse. Biotechnol. Bioeng..

[31] Hassiotis S (2014). Disease stage determines the efficacy of treatment of a paediatric neurodegenerative disease. Eur. J. Neurosci..

[32] Liao G (2007). Cholesterol accumulation is associated with lysosomal dysfunction and autophagic stress in Npc1^−/−^ mouse brain. Am. J. Pathol..

[33] Baudry M, Yao Y, Simmons D, Liu J, Bi X (2003). Postnatal development of inflammation in a murine model of Niemann-Pick type C disease: immunohistochemical observations of microglia and astroglia. Exp. Neurol..

[34] Santiago-Mujica E (2019). Hepatic and neuronal phenotype of NPC1(^−/−^) mice. Heliyon.

[35] Qiao L, Yang E, Luo J, Lin J, Yan X (2018). Altered myelination in the Niemann-Pick type C1 mutant mouse. Histol. Histopathol..

[36] Cornford EM (2016). Non-invasive gene targeting to the fetal brain after intravenous administration and transplacental transfer of plasmid DNA using PEGylated immunoliposomes. J. Drug Target.

[37] Shi N, Zhang Y, Zhu C, Boado RJ, Pardridge WM (2001). Brain-specific expression of an exogenous gene after i.v. administration. Proc. Natl. Acad. Sci. USA.

[38] Raffy S, Teissie J (1999). Control of lipid membrane stability by cholesterol content. Biophys. J..

[39] Rivest V (2007). Novel liposomal formulation for targeted gene delivery. Pharm. Res..

[40] Skjorringe T, Gjetting T, Jensen TG (2009). A modified protocol for efficient DNA encapsulation into pegylated immunoliposomes (PILs). J Control Release.

[41] Barber RD, Harmer DW, Coleman RA, Clark BJ (2005). GAPDH as a housekeeping gene: analysis of GAPDH mRNA expression in a panel of 72 human tissues. Physiol. Genomics.

